# A computational validation for the health concept maturity levels questionnaire

**DOI:** 10.3389/fpsyg.2025.1555014

**Published:** 2026-01-26

**Authors:** Arthur Trognon, Islem Habibi, Hamza Altakroury, Loann Mahdar-Recorbet, Alizée Poli, David Servais, Julien Lambert, Mathias Béjean, Denis Abraham

**Affiliations:** 1 Association Innov’Autonomie, Ecole Des Mines, Nancy, France; 2 CLINICOG, Nancy, France; 3 Consortium DynSanté, Paris, France; 4 Forum des Living Labs en Santé et en Autonomie (LLSA), Paris, France

**Keywords:** concept maturity level health, medical technology, computational psychology, computational psychometrics, user-centered research, psychological validation

## Abstract

**Background:**

The healthcare market is undergoing rapid transformation, requiring the integration of user needs from the earliest stages of product and service design. Living Labs are emerging as a model for the co-creation and evaluation of user-centered innovations. In this work, we developed a Health Concept Maturity Levels grid and questionnaire to assess the maturity of health concepts.

**Methods:**

The research process included multiple stages, starting with the creation of the Association Innov’Autonomie – Health Concept Maturity Levels Questionnaire – 178-items (CMLH questionnaire), designed to evaluate health concept maturity levels. Speech acts from Health Concept Maturity Levels expert interventions were then annotated and used as data for our machine learning and deep learning models. We used the CatBoost algorithm in the first experiment to discern individual Health Concept Maturity Levels factors from speech acts to generate factor probabilities used to feed a neural network trained to take the final decision, to evaluate whether the network could accurately identify the membership factors of Health Concept Maturity Levels criteria when presented with items from the CMLH questionnaire, thus establishing computational semantic validity.

**Results:**

The results of the study indicate that only the models trained with the true factors are able to correctly identify the corresponding factor in the sequentially encoded texts, with the exception of the Need domain’s sensitivity metric, which showed artefactual performance. The general performance of the different CatBoost algorithms used to predict one factor versus the other two showed similar performance. For the questionnaire, the models trained with the real factors also showed better performance in identifying the matching factors compared to the random factors. A marginal difference was observed between the “Need” and “Technology” factors.

**Conclusion:**

This study introduces computational semantic validity as a novel complementary approach to traditional psychometric validation, providing evidence that supports both convergent and content validity for the CMLH questionnaire. This computational method demonstrates semantic alignment between expert discourse and questionnaire structure through machine learning and deep learning techniques. However, overlaps between “Programmatic” and “Need” factors indicate a need for improvement in the Concept Maturity Levels Health model. Future work will focus on enhancing these models and investigating their potential application as a complementary validation method for other psychometric tools.

## 1 Introduction

The healthcare market is undergoing a rapid transformation, driven by technological advances and growing user expectations. Historically, technological innovation has been the primary driver behind the development of new products and services. However, it is now increasingly clear that the matching of user needs with proposed solutions is essential to ensure the acceptance, adoption, and long-term success of these innovations (Dabbs et al., 2009; Laukkanen, 2007). This market evolution highlights the need to integrate user needs from the earliest stages of the design and development of healthcare products and services (Claudy et al., 2015; Fotis et al., 2023).

This user-centered paradigm has given rise to new innovation models and necessitated robust tools for assessing concept maturity – tools whose validation, as we demonstrate here, requires innovative approaches when traditional methods prove impractical.

Living Labs (Leminen et al., 2012) have emerged as a promising model for addressing the importance of taking user needs into account in the development of healthcare products and services (Zipfel et al., 2022). This model is characterized by a user-centered, co-creation approach, where end-users, researchers, companies, and decision-makers collaborate to identify, develop and evaluate innovative solutions (Béjean et al., 2021). This approach aims to ensure that innovations truly meet users’ needs and are adapted to the contexts in which they will be deployed, thus contributing to wider adoption and dissemination (Borda et al., 2024; De Witte et al., 2021). However, the ecosystem as a whole remains fractured, and a need for readability of the intrinsic properties of projects has emerged consensually among MedTech ecosystem players (EIT Health, 2024; Hughes, 2024).

The Health Concept Maturity Levels model (CML Santé™; CMLH) was devised to provide a more inclusive and structured pathway in response to these challenges. Grounded in the Technology Readiness Levels (TRL; Mankins, 1995) and Concept Maturity Levels (CML; Wessen et al., 2013) frameworks traditionally applied to technology-driven institutional projects, the CMLH model places user needs, patterns of use, and scientific evidence on an equal footing with project considerations and technological advancement. It is therefore organized around three principal factors: Needs, Technology, and Programmatic. The Needs factor assesses the understanding of intended uses, market dynamics, and requisite clinical evidence; the Technology factor monitors the maturity of hardware-software development, data governance, and intellectual property management; and the Programmatic factor examines the robustness of project management, regulatory compliance, and financial modelling. Together, these dimensions reflect the three main stakeholder groups of the Living Lab ecosystem: end users (Needs), engineers and scientists (Technology), and industry partners or funders (Programmatic). In keeping with its conceptual predecessors, the model is formalized across nine successive levels, progressing from the initial formulation of an idea (CMLH 1) to the post-industrialization monitoring of the solution (CMLH 9).

Following this work, the CMLH grid (Béjean and Siqueira, 2019) has been developed to formalize the CMLH framework to further assess and guide the development of innovations in the healthcare sector. This grid takes into account technological, programmatic and, above all, users need to assess the maturity and relevance of innovations. The main objective of this grid is to provide a framework for identifying gaps and areas for improvement, and to facilitate the decision-making process for players involved in the selection, development or commercialization of innovative healthcare solutions.

Designed to quickly and accurately measure the maturity of healthcare concepts, the grid examines the CMLH canonical three main domains: technological maturity, programmatic maturity, and consideration of user needs. Each of these axes is then broken down into sub-factors to provide a more in-depth and nuanced analysis of concept maturity. Within the technological maturity domain, sub-factors include technology development, data management and intellectual property. For programmatic maturity, sub-factors include project management, regulatory and financial aspects. Finally, the axis of needs awareness is divided into sub-factors such as usage, market analysis and clinical evidence. The tool is made up of several questions that address these axes and sub-factors, enabling players involved in the development and commercialization of innovative healthcare solutions to better understand the strengths and weaknesses of their project, and identify potential areas for improvement. The results of the questionnaire provide an overall view of concept maturity, facilitating informed decision-making and the prioritization of resources to maximize the impact and success of innovations in the healthcare sector.

In this article, we present an experiment to validate the Association Innov’Autonomie - Health Concept Maturity Levels Questionnaire – 178-items (CMLH Questionnaire), a tool based on the CMLH grid, which assesses the maturity of health concepts according to specific criteria and supports informed decision-making by project leaders and funders.

In general, the concept of psychometric validity encompasses three major aspects: construct, content and criterion validity (Gonzalez et al., 2021; Messick, 1989; Schmeiser et al., 2006). Construct validity determines the extent to which the proposed test can identify the construct being measured. It is generally obtained by correlating the measure with a number of other measures whose correlation patterns are theoretically predictable (Westen and Rosenthal, 2003). In contrast, to construct validity, content validity verifies the representativeness of the items in a given instrument in relation to the construct under study, as well as the degree to which this instrument possesses an appropriate sample of items for the measurement of the construct under analysis (Shi et al., 2012). Finally, criterion validity aims to assess the performance predicted by the measurement tool. It is supported in particular by predictive validity, which seeks to verify the test’s performance with regard to the criterion taken as the object of study, for example by predicting a diagnosis from the set of variables (i.e., items) considered (Taherdoost, 2016).

Recently published best-practice recommendations for questionnaire validation suggest that, in validation studies, a sample size of at least 10 participants per questionnaire item is expected (Boateng et al., 2018; Nunnally, 1967). More generally, simulations have shown that at least 300–450 responses are expected in order to perform appropriate factor analyses (Guadagnoli and Velicer, 1988). In addition, it is recommended to use cross-sectional rather than longitudinal data (Boateng et al., 2018; Chesney et al., 2006).

In the present case, it did not seem realistic to be able, at a given point in time, to collect enough data to carry out a validation by conventional methods: indeed, by superimposing available data from the literature, a sample-size of 450–1780 (10:1 ratio) respondents would have been necessary. However, only 287 companies would theoretically have been able to respond to the questionnaire at its highest maturity levels (Insee, 2023), thus limiting the interpretability of the results to only the weakest maturity measures. In addition, as the data was collected at the pace of MedTech events, we would not have been able to obtain cross-sectional data, thus limiting the generalizability of the results.

Therefore, in order to overcome these limitations, we decided to carry out a computational psychometrics experiment, an analytical framework inspired by the rationale of experimental design in animal research, and based on the comparative reading of algorithmic predictions to control the informational component of certain groups of variables that may represent latent factors or psychological constructs (Trognon et al., 2022). This framework has already been used to perform construct validity and criterion validity in quantitative data (Trognon et al., 2022; Trognon and Richard, 2022).

In the present work, we have adapted the usual computational psychometrics methodology to our limitations and aimed to build a computational content validation procedure using verbal material obtained in contexts of expert assessments and coaching sessions based on the CML Health model. These corpora were transcribed and sequenced, then annotated by assigning a criterion to each speech act produced by the interlocutors. These data were then used to train a hybrid pipeline composed of a layer of CatBoost (Dorogush et al., 2018; Prokhorenkova et al., 2018) algorithms and a layer of artificial neural networks to predict the respective attribution factors of the CMLH questionnaire items, thus making it possible to verify the extent to which these items are representative of the concepts they measure. In keeping with the canonical presentation of computational psychometrics, we constructed a negative control experiment to ensure that the performance measures we performed truly represented validity evidence and not over-fitting on noise. We thus created a second experiment in which we randomly and equiprobably assigned factors to items in the corpus, making it possible to verify the impossibility of predicting the questionnaire’s attribution factors when the data are not encoded in accordance with the grid of the CMLH model.

## 2 Methods

### 2.1 Model

The French CML Health (CMLH) model (Béjean et al., 2021; Trognon et al., 2023) is a nine levels reading grid that iteratively breaks down the innovation process into three interconnected axes: needs, technology, and programmatic. It is a direct descendant of the original CML model developed by the American National Aeronautics and Space Administration (Wessen et al., 2013), which incorporated the last two domains but now includes an additional user-centered axis. Furthermore, it specifies the technology and programmatic domains to adapt them to French and European regulatory requirements in research methodology and data management (Table 1).

**Table 1:** Factorial structure of the French CML health model from Trognon et al. (2023).

Domain	Sub-domain
Needs	Usage
	Market
	Clinical proofs
Technology	Technical development
	Data management
	Intellectual property
Schedule	Project management
	Regulation
	Funding

The first axis of the CMLH model (Table 2) is derived from the Technology-Readiness Level model (Mankins, 1995). It evaluates the development of technological concepts, product management, and ownership through three formalized axes: technological development, data management, and intellectual property. The technological development axis gradually assesses processes on a scale of 1 to 9, starting from evaluating the current state of the art through critical functionality simulations, up to managing the product life cycle. The second axis focuses on how project leaders will handle data from their own devices, encompassing R&D data, protection protocols, and automation of product life cycle data. Lastly, the intellectual property axis provides insights into competitive analysis, monitoring existing patents, and managing potential infringements.

**Table 2:** Example of the different maturity levels for each sub-area of the “technological maturity” domain adapted from Trognon et al. (2023).

CML level	CML1	CML2	CML3	CML4	CML5	CML6	CML7	CML8	CML9
Technological development	Evaluation of state-of-the-art	Conceptualization and theoretical analysis	Functional simulation and testing	Creation of software demonstrator	Development of alpha prototype	Technological analysis for improvement	Automation of function testing	Addressing software bugs and issues	Management of product lifecycle
Data management	N/A	Collection of R&D data	Organization of software data	Cybersecurity measures	Availability of data	Utilization of clinical data Intellectual property of clinical data	Access to data servers	Implementation of data collection devices	Generation of material-epidemiology data
Intellectual property	Monitoring of patents	Patents pending approval	Specific granted patents	Ensuring freedom of operation	Specific granted patents	Ensuring freedom of operation	N/A	N/A	Competitive intelligence

The second axis of the CML Health model (Table 3) represents the first adaptation of the CML model developed by the American National Aeronautics and Space Administration. It incorporates project management and regulatory aspects, but in this specific case, tailored to European and French requirements for devices (such as access to the market under CE mark certification) and research. The aim is to adhere to Good Clinical Practices (GCP; see ICH-GCP, 2025) and ethical principles safeguarding individuals participating in research, protecting them from potential risks associated with acquiring new biological or medical knowledge. It should be noted that research is conducted on healthy or sick volunteers with the intention of advancing knowledge in the biological or medical fields. The French regulatory framework is based on European regulations, with recent updates for innovation seeking medical device status (ANSM’s proposed “clinical investigations” categories; ANSM, 2023). Furthermore, it ensures that methods for collecting and processing health data comply with the General Data Protection Regulation (GDPR; CNIL, 2023; Loi n° 78-17, 1978; LOI n° 2018-493, 2018) and French reference research methodologies (MR-00X; CNIL, 2024a, 2024b; Délibération n° 2015-256, 2025; Délibération n° 2018-153, 2018; Délibération n°, 2015; *Études non interventionnelles de performances concernant les dispositifs médicaux de diagnostic in vitro*, s. d.), which range from levels 1 to 3.

**Table 3:** Example of the different maturity levels for each sub-area of the “programmatic maturity” domain adapted from Trognon et al. (2023).

CML level	CML1	CML2	CML3	CML4	CML5	CML6	CML7	CML8	CML9
Project management	Identifying the driving factors	Conducting initial project risk analysis	Setting up test beds	Identifying complementary skills	Creating a detailed development plan	Updating project elements and addressing risks	Identifying marketing and sales skills	Finalizing and closing the project	Reviewing industrial development partnerships
Regulation	Establishing regulatory framework	Ensuring compliance with RGPD	Analyzing product risks	Assessing ethical aspects of the product	Collecting regulatory data	Consolidating the technical file	Compiling the CE mark file	Defining regulatory framework for data use	Renewing the CE marking
Funding	Identifying potential funding sources	Preparing the business plan	Planning financing for project demonstrators	Formalizing the business plan	Developing financial models	Establishing a minimum viable business model	Initiating series a capital raising	Updating economic assumptions with real-life data	/

The Programmatic axis of the CMLH model thus assesses the programmatic maturity of the project across three areas: project management, regulatory aspects, and financial aspects (Table 3). The project management axis evaluates the project consortium, from pilot identification to development partnership renewal, including the creation of Test Beds, and examines the nature of partnerships formed. The regulatory axis assesses programmatic maturity from the initial legal investigation surrounding the project to CE mark renewal, encompassing product risk analysis, compliance with European (e.g., MDR, GDPR; European Regulation 2017/745, 2017) and French (i.e., ethics, clinical investigations for medical devices) regulatory constraints. Lastly, the financing axis allows for gradual evaluation of financial aspects, ranging from identifying potential financing sources to updating business economic assumptions based on real-life usage data of the device.

Finally, the last axis of the CMLH model represents the true innovation of the consortium by specifying the CML model as described by NASA. It incorporates elements of consumer behaviour theory (Claudy et al., 2015), particularly addressing the barriers to innovation, and enables the evaluation of maturity in terms of needs across three axes: uses, market, and clinical evidence (Table 4). The Uses axis provides insights into the device’s value and ensures user-centric product development in terms of uses. It assesses development from identifying the social context and public health implications to evaluating the perceived quality of care through patient evaluation methods (PREM). This axis verifies the elimination of certain functional barriers, such as conflicts with established usage patterns. The Market axis examines the competitive landscape concerning device uses, including market literature reviews and evaluations of market segment diversity and respective access strategies. This axis confirms the elimination of functional value barriers and verifies the uniqueness of the device’s value proposition. Finally, the Clinical Proof axis assesses the quality of clinical investigations conducted on the device, ranging from comprehensive literature analysis to formalized processes for evaluating the perceived quality of device results by patients (PROM). This axis addresses the functional barrier of uncertainty that arises when end-users have limited access to devices under development, as described by Stone and Grønhaug (Stone and Grønhaug, 1993).

**Table 4:** Example of the different maturity levels for each sub-area of the “need maturity” domain (PREM: patient reported experience measure; PROM: patient reported outcomes measure) adapted from Trognon et al. (2023).

CML level	CML1	CML2	CML3	CML4	CML5	CML6	CML7	CML8	CML9
Usage	Understanding social and public health context	Identifying practice situations that justify the need	Collaboratively developing tailored usage scenarios	Conducting UX/UI lab evaluations	Defining the usage industrialization scheme	Assessing usability and acceptability	Evaluating ecological impact of a pre-series	Examining real-life organizational impact	Ensuring quality control of patient reported experience
Market	Reviewing the existing market literature	Identifying the value proposition	Defining product positioning and expected impact	Quantifying the expected impact	Developing market access strategy	Characterizing the device based on usage surveys	Implementing marketing elements (deployment, export)	Refining go-to-market strategies by customer type	Marketing across different markets
Reviewing relevant clinical literature	Review of the clinical literature	Identifying the medical need	Formulating the clinical strategy	Initiating preliminary clinical trials	Analyzing results from clinical trials	Drafting study reports (publications)	Conducting multi-center clinical trials	Performing medico-economic studies	Ensuring quality control of patient reported outcomes

### 2.2 Subjects

Participant data were collected during sessions organized between 2021 and 2023, including auditions of innovative project leaders in the field of medical technologies (MedTechs). In chronological order, the data came from two individual semi-directive interviews carried out as part of the preparation of the methodological deployment; five consortium auditions organized during the Future4Care 2022 start-up competition; three semi-directive group interviews carried out during the French National Digital Health Sector’s Call for Expressions of Interest; and a day of presentations (symposium) as part of the e-Meuse Expert Committee meeting, whose aim was to identify the optimal conditions for the deployment of digital innovation in healthcare in eastern France.

All participants received detailed information on the objectives and purpose of the study, and ethical consents were obtained online in agreement with the Declaration of Helsinki. The study protocol was approved by the Institutional Review Board Commission Nationale de l’Informatique et des Libertés (registration no. 2,230,503).

### 2.3 Study design

Verbatim from pitches, individual and group interviews were recorded, then transcribed according to the formalism of the 2TK model (Trognon, 2022; Trognon et al., 2025), that is, transcribed into speech acts sequenced by the pauses identified in the discourse in order to reproduce its kinetics as closely as possible, as well as to identify the most circumscribed packets of information possible. Each speech act was then labeled according to three parameters: the factor (three levels: need, programmatic, technology; otherwise “Null”); the sub-factor (three levels per factor: market, uses, clinical evaluation for the “Need” factor; technological development, intellectual property, data management for the “Technology” factor; and financing, regulatory aspects, project management for the “Programmatic” factor); and the CMLH level (nine levels: CML1 to CML9). A total of 10,952 speech acts were labeled; then only non-Null speech acts (*n* = 2,070) were retained.

These speech acts were used to build a two-layer computational pipeline with two complementary and distinct objectives: a first “expert systems” layer of CatBoost (Dorogush et al., 2018; Hancock and Khoshgoftaar, 2020; Prokhorenkova et al., 2018) algorithms, comprising a per-factor algorithm for calculating the probability of a speech act carrying information about a given CMLH factor or belonging to another factor or noise; and a second layer consisting of an artificial neural network embedding the probability of each expert system and providing a final decision regardless of the textual material provided, which was used to predict the membership factor of the questionnaire items from which the speech acts had been labeled.

### 2.4 Scale development and item generation

The items were developed directly from the CMLH grid of the Forum Living Lab Santé Autonomie (version 2021/12; Supplementary Data Sheet 1). We generated 1 item for each criterion, designed to elicit a personal representation of the project’s status, from which the participant could make a judgment between the induced representation and the subjective perception of progress. A total of 178 items were generated from this grid (French: Additional File 2; English: Additional File 3).

We chose to base the questionnaire on an Osgood-type scale, constructing the lower and upper bounds in a logic of continuity, where items belonging to higher levels have lower bounds that can be superimposed to some extent on the upper bounds of lower items. Similarly, we chose to scale the questionnaire on a linear 6-point scale in order to avoid neutrality bias, although its existence is still debated (Lozano et al., 2008; Weijters et al., 2010). In addition, the stimuli were built around first-person personal pronouns, which allows for greater immersion of the subject in relation to the situational context about which he or she is being questioned (Hartung et al., 2016), which seems particularly appropriate in the case of a maturity assessment of project concepts.

However, not all sentences describing criterion within the original grid were unambiguous in terms of their specific factors, necessitating few tuning editions to ensure their alignment with the intended factor. For instance, the phrase “The technical tools that can meet the *needs* are clearly identified” has the factor “Technology” in the questionnaire, even though it contains the word “need.” In this case the criterion was modified to “The appropriate tools to meet the technical requirements are clearly identified.” in the scale. Once the item adaptation was completed, the corpus underwent annotation and classification was performed using the computational pipeline described in the relevant section.

### 2.5 The computational psychometrics paradigm

Computational psychometrics is a group of innovative procedures that uses machine learning techniques to assess the validity of measurement instruments, scales or questionnaires. This approach verifies whether the instrument’s items or questions adequately reflect the concept they are designed to measure (Trognon et al., 2022).

In our approach to evaluate the computational semantic validity of the CMLH questionnaire, we employed a hybrid machine-learning/deep-learning pipeline within the computational psychometrics framework. This analytical paradigm, inspired by the experimental design rationale used in animal studies, uses algorithmic predictions to control the informational overlap among groups of variables representing latent psychological constructs (Trognon et al., 2022). By structuring our experiments with positive and negative controls, we ensure that the observed effects are not merely random fluctuations, but reliably reflect the model’s capacity to identify the underlying dimensions of the CMLH questionnaire.

In this context, the experimental condition correspond to the real factor annotations, whereas negative controls involve random factor assignments, thus measuring the experiment’s background noise. To predict the membership factor (i.e., “Need,” “Programmatic,” or “Technology”) from a corpus of labeled speech acts and questionnaire items, we split our dataset into training and test sets. We then trained a CatBoost classifier (an ensemble-based algorithm well-suited to handling categorical features) on the training set. Each classifier was subsequently tested via cross-validation on the held-out data to measure accuracy, sensitivity, and specificity. Finally, an Analysis of Variance (ANOVA) was conducted to compare performance metrics across our experimental conditions (real factor annotations vs. random assignments), in order to confirm that the models grounded in the actual CMLH factors performed significantly better than those operating on random or mismatched annotations.

### 2.6 Computational semantic validity

Within the framework of computational semantic validation, an algorithmic model is trained on a portion of verbatim data obtained from discussions focused on project maturity, and its performance is then evaluated on a different portion of the data (called a test set). The aim is firstly to demonstrate that the model can reliably and accurately predict the dimensions or factors underlying the discourse and superimposable on the CMLH questionnaire criteria from the words spoken by the participant on a given sample, thus providing evidence for the factorial structure (supporting construct validity); then to demonstrate that this model is capable of correctly identifying these dimensions in a new data set (i.e., the questionnaire), establishing semantic convergence between expert language and item content (supporting content validity). This computational semantic validity bridges construct and content validation by verifying both the structural integrity of factors and the semantic appropriateness of items through natural language analysis. This approach thus offers a new computational complement to traditional validation methods, providing convergent evidence that items capture both the theoretical structure and the authentic language of the domain. Unlike traditional content validation which relies on post-hoc expert judgment, computational semantic validity can be implemented during the development phase, offering empirical evidence of semantic alignment before formal pre-testing.

### 2.7 Factorial structure and semantic validation

The method employed in this study aims to circumvent the limitations inherent in classical psychometric validation by using a computational approach. Here’s how it could help overcome these constraints while providing relevant information on factor structure, internal consistency and reliability.

*Factorial structure*: Using a machine learning model, we are able to identify which factors (in our case, the categories of ‘Technology’, ‘Programming’ and ‘Need’) are relevant to each statement. This is an indirect way of exploring the factor structure of the dataset. Indeed, if the model is able to predict these factors correctly, it suggests that they are well-defined and have real meaning in the context of technological innovation in healthcare.

*Internal consistency and reliability*: Internal consistency and reliability are assessed through the performance of the machine-learning model. In this context, internal consistency is reflected by the model’s ability to make consistent predictions on different parts of the training dataset. Reliability, on the other hand, is assessed by examining the model’s performance on a novel test dataset. If the model makes accurate and consistent predictions on this data set, this suggests that it is reliable.

This approach has the advantage of not requiring a large number of participants, which is a major constraint of classical psychometric validation. What’s more, the machine learning model can be re-trained as new data are collected, enabling continuous improvement in accuracy and reliability.

It’s important to note that this computational approach is not a substitute for conventional psychometric validation. On the contrary, it offers a practical alternative when the data collection required for conventional validation is not feasible. In addition, it allows conclusions to be drawn from a fundamentally smaller data set, but one that is potentially richer in information, offering the possibility of continuous updating as new data is collected. It can also be superimposed on conventional validation techniques, providing a new body of evidence for the validity of the measurements produced by these tools.

### 2.8 Computational semantic validation procedure

Data consisted of transcribed text derived from meetings and presentations inscribed by 2TK method (Trognon, 2022; Trognon et al., 2025). For performing the analysis, three experts assigned labels to the transcribed phrases based on the relevant factors from the CML Health model (Need, Programmatic and Technology). For representing the transcribed phrases in a format suitable for analysis, the Bag of Words technique was applied (Maron et al., 1959; Qader et al., 2019). This approach transformed the text into numerical vectors, enabling subsequent computational processing and modelling.

**Figure 1:**
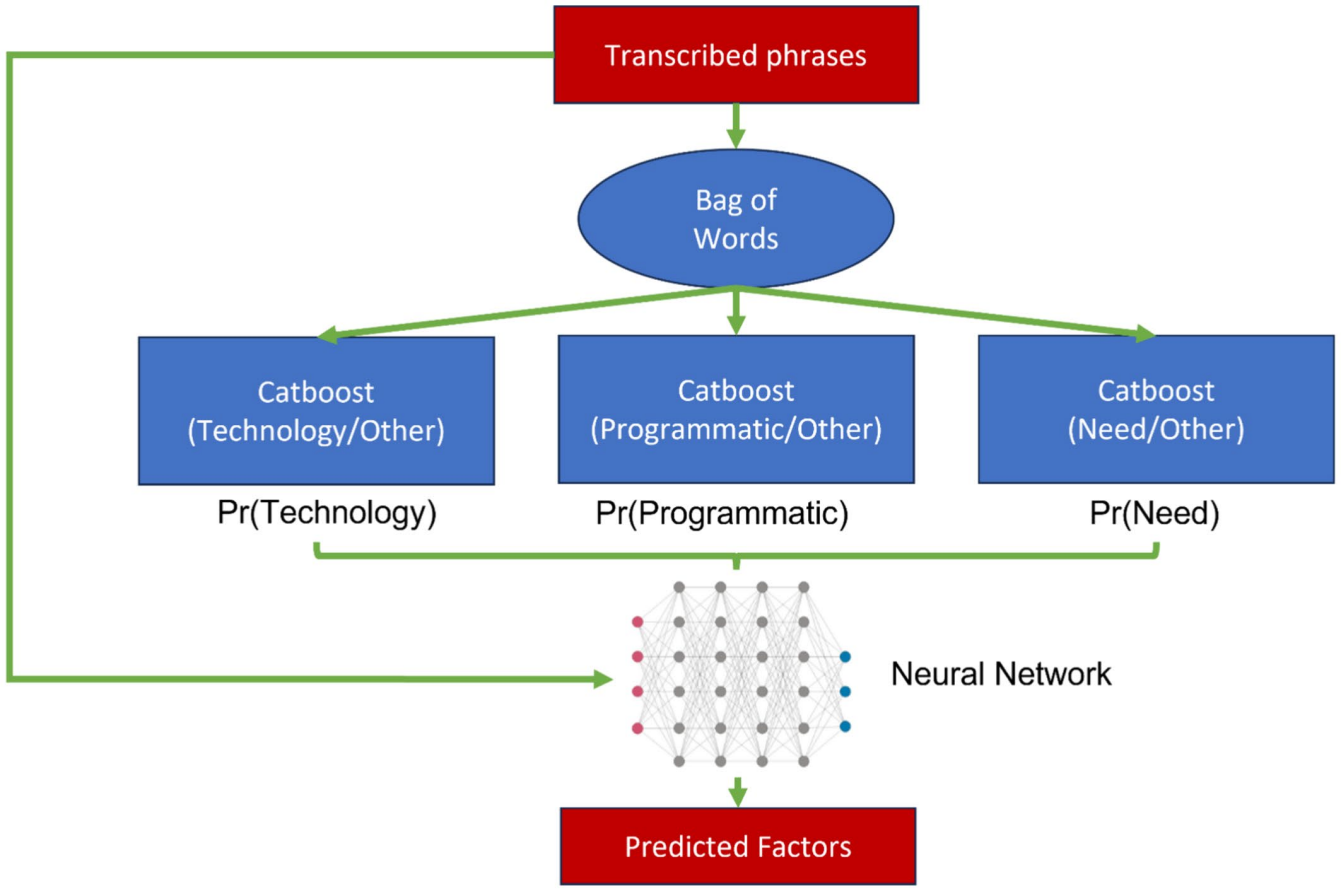
Computational analysis pipeline. For detailed neural network architecture specifications including layer dimensions, activation functions, and training parameters, see Section 2.11.

The proposed model in this work was to have two layers of classification (as shown in Figure 1): a layer which provides a probability that transcribed phrases belong to a certain factor in the CML metric; while the aim of the second layer is to provide the predicted label of the transcribed phase based on the given probabilities and the corresponding transcribed phrase.

To optimize the performance of the Catboost classifier, the hyperparameters were fine-tuned using Bayesian Optimization. This optimization approach ensured that the hyperparameters of the classifier were optimized to obtain improved classification results. These hyperparameters (and their corresponding ranges) were: The number of estimators ([100, 1,500]), the learning rate ([0.01, 0.3]), the depth of each decision tree ([4, 10]), the L2 regularization coefficient ([1, 10]), the randomness strength ([0, 1]), the temperature parameter for the bagging process ([0, 10]) and the border count parameter for categorical features ([32, 255]). Furthermore, the number of initial points for the Bayesian Optimization was set to five and the number of iterations was set to 20. Subsequently, the Catboost classifier was utilized to generate probabilities for each category [Pr(Need), Pr(Programmatic) and Pr(Technology)] associated with the transcribed text. These probabilities served as an indication of the likelihood of each category’s presence in the transcribed phrases.

Furthermore, in order to enhance the classification process, a Neural Network was trained using the probabilities generated by the Catboost classifier, in addition to the transcribed text. Another set of data was reserved for testing the performance of this Neural Network layer.

### 2.9 Data preprocessing

Two annotated spreadsheets served as the starting point: the full corpus of 10,952 speech acts and a subset of 2070 non-null acts. Each file was randomly partitioned: 30% was sequestered as a hold-out set using a fixed seed (42) and the remaining 70% from both files was merged, producing a training corpus of 9,115 utterances. These strings were first cleansed of a transcription artefact (the leading token “texte”) via a regular-expression substitution, then converted to lower case. Tokenization (Webster and Kit, 1992), French stop-word removal, and Porter stemming (Porter, 1980) were performed in a single pass with nltk (Bird and Loper, 2004). The cleaned tokens were finally translated into a Bag-of-Words representation through scikit-learn’s CountVectorizer (Pedregosa et al., 2018), restricted to unigrams and capped at the 350 most frequent terms to limit sparsity; the resulting dense term-document matrix therefore measured 9,115 × 350. In parallel, each utterance inherited four one-hot factor flags (Need, Programmatic, Technology, Null) as well as a single mutually exclusive multi-class label directly reflecting the expert annotation. All text preparation and feature extraction were carried out in Python 3.11.13 with pandas 2.2.2, nltk 3.9.1 catboost 1.2.8, numpy 2.0.2 and scikit-learn 1.6.1.

### 2.10 Cross-validation procedure

For model evaluation, we employed 5-fold cross-validation using scikit-learn’s k-Fold implementation (v1.6.1), following established best practices for machine learning validation in limited sample contexts (Hastie et al., 2005; Vabalas et al., 2019). The choice of 5-fold validation represents a balance between computational efficiency and variance reduction, particularly appropriate for datasets with moderate sample sizes (Rodriguez et al., 2009). The data was randomly partitioned into five equal-sized folds without stratification, as the relatively balanced distribution of factors in our dataset (Need: *n* = 690, Programmatic: *n* = 689, Technology: *n* = 691) did not necessitate stratified sampling. Each fold served once as the test set while the remaining four folds were used for training, ensuring that all speech acts were used for both training and testing exactly once. For each fold, we computed accuracy, sensitivity, and specificity metrics. The reported results represent the mean performance across all five folds, with standard deviations indicating cross-fold variance. The random seed was set to 42 for reproducibility.

### 2.11 Artificial neural network architecture

The neural network component of our pipeline was implemented using Keras with TensorFlow backend. The architecture consists of a feedforward neural network with the following specifications: an input layer accepting three-dimensional vectors corresponding to the probability outputs from the three CatBoost classifiers [Pr(Need), Pr(Programmatic), Pr(Technology)], followed by a single hidden layer containing 64 neurons with Rectified Linear Unit (ReLU) activation function, and an output layer with 3 neurons (corresponding to the four possible classes: Need, Programmatic, Technology) using softmax activation for multi-class classification. The network was trained using the Adam optimizer with categorical cross-entropy as the loss function. Training was performed for 10 epochs with a batch size of 32, using 20% of the training data for validation. The train-test split was set at 70–30% with a fixed random seed (32) to ensure reproducibility. This relatively simple architecture was chosen to prevent overfitting given our dataset size while maintaining sufficient capacity to learn the mapping between CatBoost probability distributions and final factor assignments.

## 3 Results

### 3.1 Model selection

**Figure 2:**
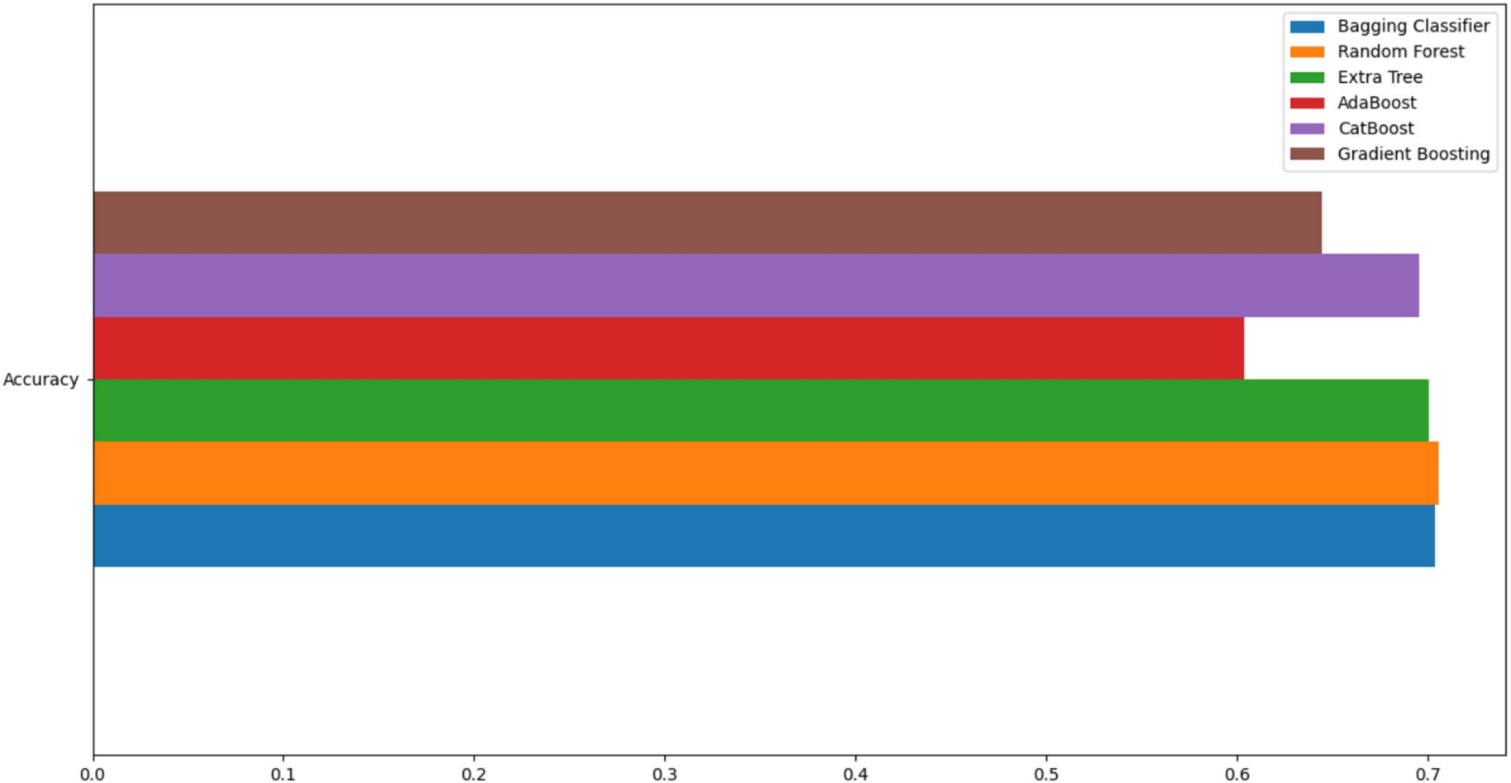
Model selection for building the computational pipeline.

Prior to evaluating the classification performance, we first determined the most suitable classifier for our study. A comparison was performed among various classifiers, including Decision Tree, Bagging classifier (comprising 500 decision trees), Extra Tree, Gradient Boosting, AdaBoost, CatBoost, and Random Forest (Figure 2).

Using a 2/3 train and 1/3 test split of the data, several classifiers demonstrated comparable high performance, with Extra Trees and CatBoost achieving the highest accuracy scores (both approximately 0.72–0.73). While Extra Trees showed marginally higher accuracy in this initial comparison, we selected CatBoost for our pipeline due to several practical advantages: its native handling of categorical features without preprocessing, its built-in support for missing values, and its robustness against overfitting through gradient boosting with ordered boosting. These characteristics were particularly relevant for our study given the mixed categorical and numerical nature of our annotated speech acts and the potential for incomplete annotations in real-world applications.

### 3.2 CatBoot “expert” algorithms train-testing

**Figure 3:**
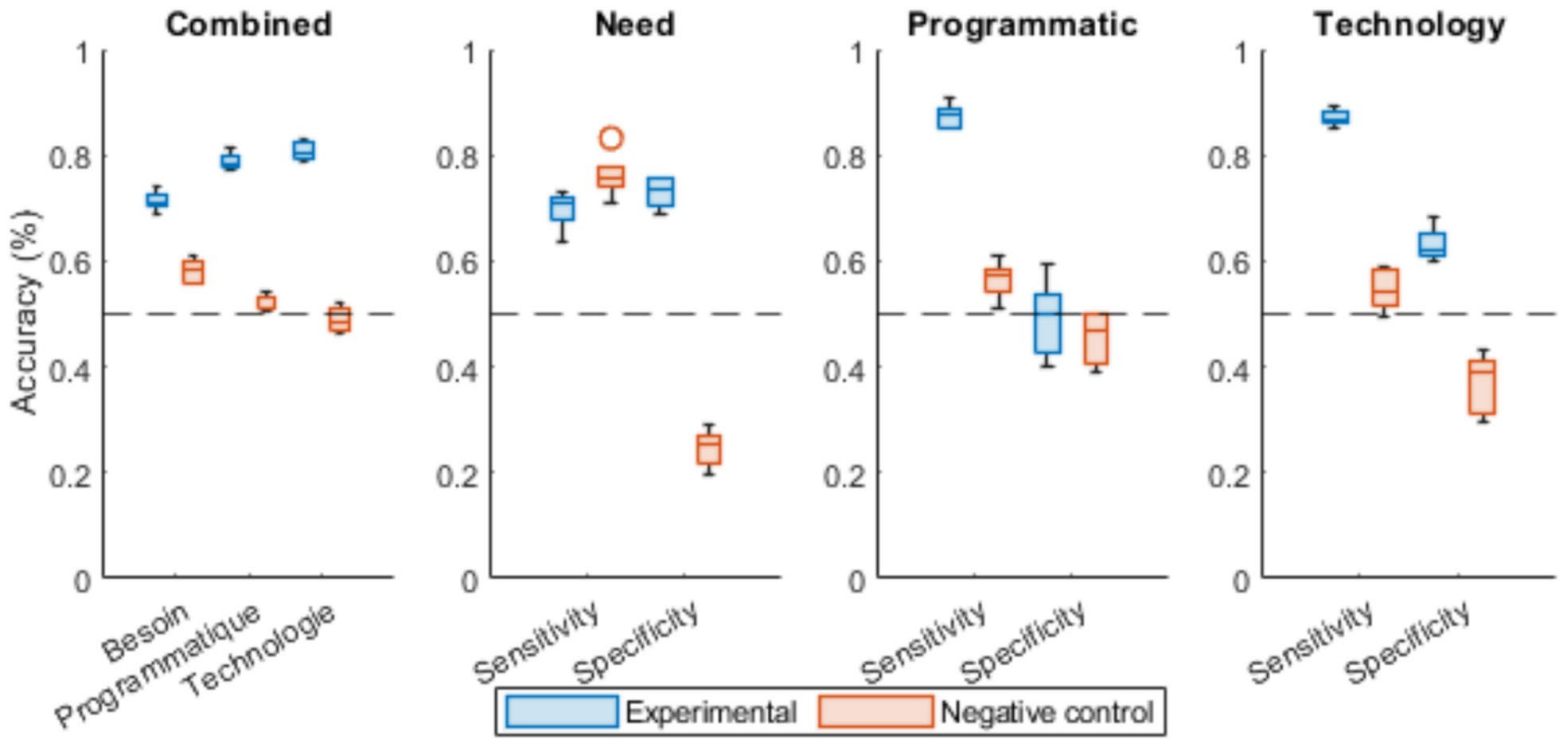
The average performance of each factor expert CatBoost in identifying the membership factor when a speech act related to the CML health model is presented for each condition in 5-fold cross-validation. Box plots display median (central line), interquartile range (box), 1.5 × IQR whiskers, and outliers (individual points). *N* = 414 speech acts per fold (2,070 total non-null speech acts divided across 5-fold) for the experimental condition with true factor labels, and equivalent sample size for the control condition with randomly assigned labels. The detailed cross-validation metrics are available in Supplementary Table S1.

Classification performance results comparing real CML Health factor labels with random ones are shown in Figure 3. The data suggest that only models trained using the real factors are able to correctly identify the matching factor based on sequentially encoded text corpora, except for the need domain whose artefactual performance was measured on the sensitivity metric.

Regarding the general accuracy measures, a two-factor ANOVA (Performance~Condition*Algorithm) revealed that the different algorithms used to predict one factor in contrast to the two others showed similar performances [*F*(2,24) = 0.606, *p* = 0.554, *η*^2^*p* = 0.05]; and the randomization of the factor for the control condition was associated with a very large performance loss [*F*(1,24) = 1122.131, *p* < 0.001, *η^2^p* = 0.98, Cohen’s *d* = 5.28]. A significant interaction with a large effect size was detected between the different CatBoosts performances and the condition [*F*(2,24) = 62.604, *p* < 0.001, *η^2^p* = 0.84].

Further post-hoc analysis revealed a significant difference between experimental and control conditions for all CatBoost algorithms [Table 5; Figure 3 (left)].

**Table 5:** Tukey post-hoc test results for the accuracy (algorithm*condition interaction).

CatBoost algorithm	Experimental vs. control (*p*-value)
“Need” identifier	<0.001
“Programmatic” identifier	<0.001
“Technology” identifier	<0.001

Concerning the detection measures, a three-factor ANOVA (Performance~Condition*Algorithm*Metric) revealed significant effects with very large effect sizes for metric [*F*(1,48) = 441.298, *p* < 0.001, *η^2^p* = 0.90] and group [*F*(1,48) = 412.299, *p* < 0.001, *η^2^p* = 0.90, Cohen’s *d* = 1.44]. Significant interactions were observed between the algorithms and the condition [*F*(2,48) = 10.435, *p* < 0.001, *η^2^p* = 0.30], metric and condition [*F*(1,48) = 10.290, *p* = 0.002, *η^2^p* = 0.18], and between condition, algorithm and the metric considered [*F*(2,48) = 122.204, *p* < 0.001, *η^2^p* = 0.84]. Further Tukey’s post-hoc analysis revealed significant differences between experimental and control conditions for all CatBoost algorithms, except for the sensitivity in the need domain and for the specificity in the programmatic domain (Table 6; Figure 3).

**Table 6:** Tukey post-hoc test results for the metrics (algorithm*condition*metric interaction).

CatBoost algorithm	Sensitivity (Exp vs. Ctrl)	Specificity (Exp vs. Ctrl)
“Need” identifier	0.45	<0.001
“Programmatic” identifier	<0.001	0.96
“Technology” identifier	<0.001	<0.001

These results may be related to the overlap between the “regulatory” sub-factor of the “programmatic” factor and the “clinical evaluation” sub-factor of the “need” factor, both of which share criteria concerning ethical questioning and subsequent evaluation by human protection committees in order to initiate clinical investigations.

### 3.3 Neural network performance on the questionnaire

**Figure 4:**
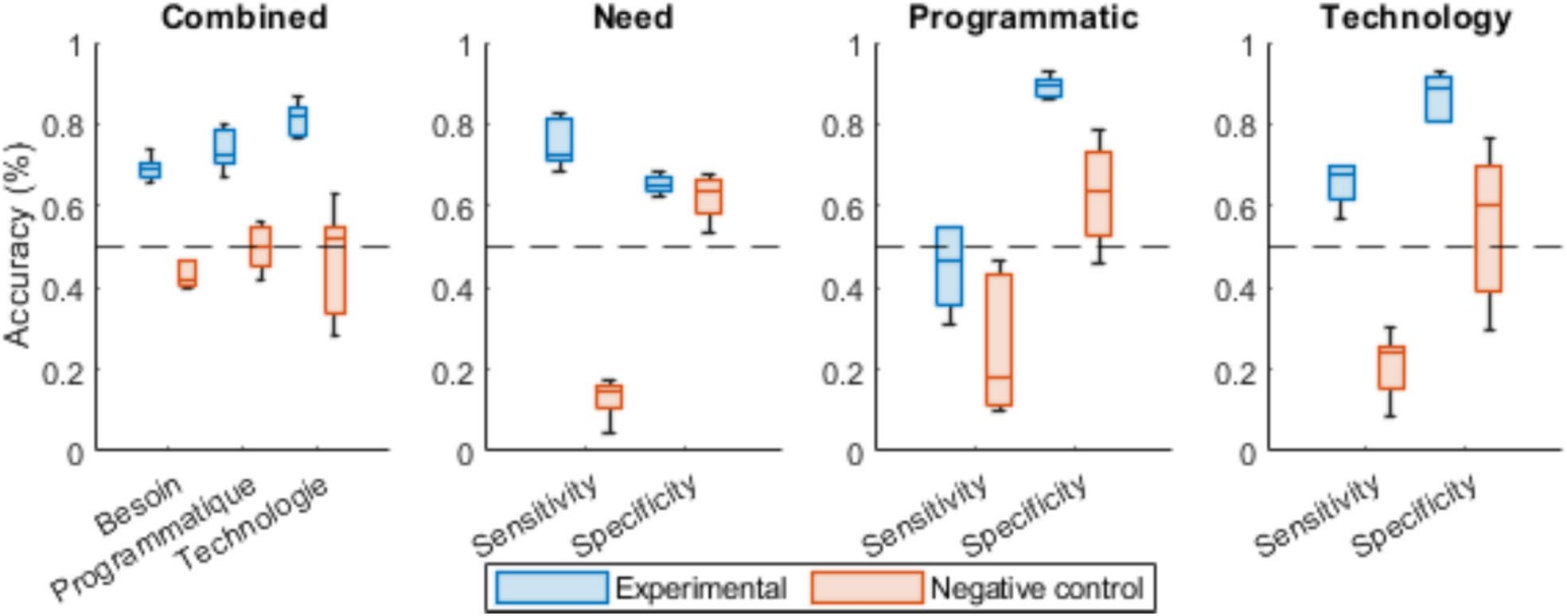
The average performance of the neural network in identifying the membership factor of each CML health questionnaire item for each condition in 5-fold cross-validation. Box plots display median (central line), interquartile range (box), 1.5 × IQR whiskers, and outliers (individual points). *N* = 36 items per factor (178 total questionnaire items, with approximately equal distribution across the three main factors: Need, programmatic, and technology) evaluated across 5 folds for both experimental (true factor labels) and control (randomly assigned labels) conditions. The detailed cross-validation metrics are available in Supplementary Table S2.

Classification performance results for questionnaire factor identification, comparing conditions with real CML health factor labels versus random ones, are shown in Figure 4. The data suggest that only models trained using the real factors are able to correctly identify the matching factor when a questionnaire item is presented.

Regarding the general accuracy measures, a two-way ANOVA (Performance~Condition*Factor) revealed that the neural network showed a medium effect for factors [*F*(2,24) = 3.237, *p* = 0.057, *η^2^p* = 0.21], with a marginal difference between the factors “Need” and “Technology” measured using post-hoc Tukey’s procedure (*p* = 0.054). As previously, a very large effect was observed in terms of performance differences between the experimental and control conditions [*F*(1,24) = 123.52, *p* < 0.001, *η^2^p* = 0.84, Cohen’s *d* = 3.68], but no significant interaction between the variables under consideration was measured [*F*(2,24) = 1.748, *p* = 0.196, *η^2^p* = 0.13].

Concerning the detection measures, the three-way ANOVA (Performance~ConditionFactorMetric) revealed significant effects with very large effect sizes for the metric [*F*(1,48) = 130.81, *p* < 0.001, *η^2^p* = 0.73, Cohen’s *d* = 1.39] and the experimental condition [*F*(1,48) = 147.593, *p* < 0.001, *η^2^p* = 0.75, Cohen’s *d* = 1.53]. Significant interactions were observed between the target factor and the metric used to assess the performance [*F*(2,48) = 5.872, *p* = 0.005, *η^2^p* = 0.20], the experimental condition and the metric used to assess the performance [*F*(1,48) = 17.591, *p* < 0.001, *η^2^p* = 0.27], and between the three variables under consideration [*F*(2,48) = 14.601, *p* < 0.001, *η^2^p* = 0.38]. Further post-hoc Tukey’s analysis confirmed as intended a significant difference between experimental and control conditions for both sensitivity (*p* < 0.001) and specificity (*p* < 0.001).

Concerning (target factor × experimental condition) interaction specifically, a subsequent post-hoc Tukey procedure revealed significant differences between sensitivity and specificity for all three target factors, suggesting that its abilities to identify and exclude different target factors are not equivalent (Table 7).

**Table 7:** Tukey post-hoc test results for the target factor × experimental condition interaction.

Target factor	Sensitivity vs. specificity (*p*-value)
Need	<0.001
Programmatic	<0.001
Technology	<0.001

Finally, the Tukey post-hoc analysis investigating the triple (target factor × experimental condition × metric used) interaction showed an opposite pattern in regard to the first experience (Table 8).

**Table 8:** Tukey post-hoc test results for the target factor × experimental condition × metric used interaction.

Target factor	Sensitivity (Exp vs. Ctrl)	Specificity (Exp vs. Ctrl)
Need	<0.001	0.99
Programmatic	0.11	0.005
Technology	<0.001	<0.001

These results again suggest overlaps between the need and programmatic factors. The pattern inversion between the two experiments is an interesting observation that may reinforce the idea of a strong overlap between the two factors.

## 4 Discussion

Using a hybrid approach involving supervised machine-learning and unsupervised deep-learning approaches, we were able to provide evidence of computational semantic validity for the Association Innov’Autonomie – Health Concept Maturity Levels Questionnaire – 178-items (CMLH questionnaire).

First, we carried out an experiment using a series of CatBoosts, state-of-the-art supervised classification algorithms that verified that it was possible to identify the Health CML factor specifically emerging from the noise when a speech act was presented.

These probabilities were then used to run an experiment using an artificial neural network, which showed that it was possible to identify the membership factors of Health CML criteria when presented with questionnaire items.

To the best of our knowledge, and in spite of the evidence provided for the validity of the questionnaire itself, we believe that this work is the first to investigate the validity of the Concepts Maturity Levels model itself, even though this model has already been built in several iterations before its French declension in the healthcare sector (Béjean and Siqueira, 2019; Mankins, 1995).

In addition, the present work could constitute the first empirical evidence that the Living Lab paradigm can be formalized into a coherent, measurable construct encompassing the full spectrum of stakeholder perspectives. By operationalizing the CMLH model across the Needs, Technology and Programmatic axes, and showing, through a hybrid machine-learning pipeline, that each axis can be detected and discriminated in authentic discourse, we provide material evidence that the conceptual structure of the Living Lab model is not merely heuristic but psychometrically tractable.

Beyond its validation value, the approach lends itself to two practical applications. First, because CMLH scores can be generated repeatedly with minimal respondent burden, they can serve as longitudinal markers during coaching or incubation programs, enabling project teams and mentors to apply growth-curve or time-series analyses to verify progression along each maturity axis and to adjust support strategies accordingly. Second, the same scores can be mapped against expert appraisal grids to give entrepreneurs granular feedback on their pitch content: highlighting domains where additional detail would strengthen credibility and flagging over-represented areas that could be streamlined for clarity. This evidence-based feedback loop promises to refine both the substance and the delivery of innovation pitches, ultimately enhancing alignment between project leaders, evaluators and end-users.

Furthermore, these works are the first to our knowledge to investigate psychometric validation through computational semantic analysis of qualitative data, introducing computational semantic validity as a novel form of evidence within the overall framework of psychometric validity. This approach uniquely demonstrates how natural language patterns from expert discourse can provide convergent evidence supporting both construct and content validity simultaneously, thus establishing a new empirical bridge between theoretical structure and domain-authentic language representation.

However, the results suggest room for improvement: indeed, the specificity and sensitivity measures for the “Programmatic” and “Need” factors were not satisfactory in the inversion experiments, suggesting overlaps between these two factors. This overlap represents a genuine theoretical issue inherent to the current CML Health model structure rather than a methodological limitation or inadequate item discrimination. This may be attributed to the items measuring the ethical dimensions of the project, which are conceptually shared by both the “Clinical Evaluation” sub-factor of the “Need” factor in the criteria evaluating clinical evidence, notably in the constitution of clinical investigation files and the analysis of clinical study data in general; and the “Regulatory” sub-factor of the “Programmatic” factor, which evaluates the ethical framework associated with clinical investigations, notably the constitution of personal protection files, as well as the planning of the clinical studies themselves.

This conceptual overlap is not artefactual but reveals a fundamental challenge in the current factorial structure of the CML Health model: ethical and regulatory aspects of clinical investigations inherently bridge both user needs (through clinical evidence requirements) and programmatic considerations (through regulatory compliance). The pattern inversion observed between the two experiments (Tables 7, 8) further supports this interpretation, suggesting that the overlap is consistent across different analytical approaches.

A re-allocation or modification of the initial grid thus seems necessary in order to increase the internal consistency of the Health CML model itself. This finding, while highlighting a limitation of the current model, represents a valuable contribution of our computational validation approach in identifying areas for theoretical refinement.

Several limitations of this study need to be acknowledged. First, the dataset used was relatively small, and the number of individual participants was limited. This restricted the opportunity for A-B testing on a fresh dataset due to the multiple splitting operations that were required in the machine learning processes. As such, our findings should be considered preliminary, and caution is required in generalizing them to other contexts.

Second, the study did not include a traditional quantitative measure on the questionnaire itself. While this hybrid machine-learning/deep-learning approach introducing computational semantic validity provides novel empirical evidence for both construct and content validity through language analysis, it is designed to complement rather than replace canonical psychometric methodologies. The absence of traditional quantitative validation in our study limits the triangulation of evidence, as computational semantic validity ideally works in conjunction with expert judgment and classical psychometric measures to establish comprehensive validity. Future studies would benefit from combining this computational approach with traditional validation methods to provide converging lines of evidence.

Third, the annotation process represents another important limitation. Due to the large volume of speech acts (*n* = 10,952) and time constraints, we employed a division-of-labor approach where three expert annotators each coded distinct, non-overlapping portions of the corpus rather than having multiple annotators code the same material. This design prevented us from calculating inter-rater reliability metrics (e.g., Cohen’s kappa, Fleiss’ kappa, or intraclass correlation coefficients), which are standard quality indicators in annotation tasks.

Moreover, a further limitation stems from our exclusive reliance on a Bag-of-Words representation. Although Bag-of-Words is transparent and computationally lightweight (Zhang et al., 2011), it deliberately discards word order, syntax, and broader discourse relations, leaving each token isolated from its context. This context-agnostic view prevents the disambiguation of polysemous terms, obscures synonymy, and fragments multi-word expressions that are semantically meaningful in the MedTech domain (e.g., “CE marking,” “Good Clinical Practice”). The resulting high-dimensional, sparse vectors can inflate noise and magnify overlap between conceptually adjacent sub-factors (e.g., the confusion we observed between the “Regulatory” and “Clinical Evidence” criteria). To mitigate these shortcomings, future iterations could incorporate more context-aware natural-language representations by enriching the feature space with systematic n-grams that would capture short collocations and domain-specific phrases or by creating semantically grounded embeddings that could replace or complement the Bag-of-Words, allowing semantically similar terms to cluster together while preserving distinctions borne of context.

Lastly, the study did not conduct a detailed analysis of the decision shifts that are to be considered in future studies. Understanding these shifts could potentially provide insights into the underlying mechanisms of how the algorithms identify the membership factors, providing information about the factorial structure as well. This may have resulted in an incomplete view of the behaviour and performance of the models used. This lack of detailed analysis of decision shifts represents an important avenue for future research on Concept Maturity Levels and psychometric tools. Future studies should aim to address these limitations to provide a more comprehensive understanding of the performance of machine and deep-learning methods in validating psychometric instruments.

## 5 Conclusion

This study validates the CMLH questionnaire through computational semantic analysis, demonstrating that questionnaire items accurately capture the theoretical constructs they measure. Beyond confirming the instrument’s validity, our approach reveals the practical potential of computational psychometrics for validating specialized assessment tools in contexts where traditional sample requirements are prohibitive. Thus, this work establishes the first validation evidence for the CMLH questionnaire as a tool for assessing healthcare innovation maturity while pioneering a methodological approach applicable to other domains facing similar validation constraints.

## Data Availability

The original contributions presented in the study are included in the article/Supplementary material, further inquiries can be directed to the corresponding author.
