# Supplementary material for: A computational validation for the health concept maturity levels questionnaire
Source: Front Psychol. 2026 Jan 26;16:1555014. doi: 10.3389/fpsyg.2025.1555014 (PMC12884647; doi:10.3389/fpsyg.2025.1555014)
Supplement: Supplementary file 3 [file Data_Sheet_1.pdf]

FORUM LLSA

	Technologie	Besoin	Programmatique
**CML 1**<br>**Formulation de l'idée**	**Développement technique**<br>T1.1 Ancrage technique du besoin<br>T1.2 Etat de l'art et concurrence<br>T1.3 Stratégie initiale, concept, éléments d'architecture des systèmes et réseaux<br><br>**Gestion des données**<br><br>**Propriété intellectuelle**<br>T1.4 Confidentialité<br>T1.5 Veille brevet	**Usages**<br>B1.1 Contexte social et de santé publique<br>B1.2 Contexte d'usage et exigences<br>B1.5 Approche populationnelle et empowerment du patient et de son entourage<br>B1.9 Environnement projet propice créativité et à l'innovation<br><br>**Marché**<br>B1.3 Problème socio-économique et médical<br>B1.6 Revue de la littérature marché<br>B1.7 Expression de besoins individuels et collectifs<br><br>**Evaluation clinique**<br>B1.4 Principe actif, modalité d'observation<br>B1.8 Revue de littérature clinique	**Gestion de projet**<br>P1.3 Planification du projet<br>P1.4 Pré-identification d'un collège d'évaluateurs<br>P1.5 Identification des contributeurs potentiels au projet<br>P1.6 Identification du pilote<br><br>**Réglementaire**<br>P1.2 Identification du cadre réglementaire<br><br>**Financement**<br>P1.1 Mise en place de l'équipe du projet<br>P1.7 Identification des sources de financement<br>P1.8 Plan de financement R&D
**CML 2**<br>**Premiers concepts, compréhension des exigences d'usage et de faisabilité**	**Développement technique**<br>T2.1 Analyse de différents principes, théorie ou simulations pour les fonctions essentielles<br>T2.3 Identification des verrous technologiques et orientation des développements techniques<br>T2.6 Sélection d'un principe privilégié<br><br>**Gestion des données**<br>T2.4 Collecte de data pour la R&D<br><br>**Propriété intellectuelle**<br>T2.5 Brevet(s) de principe	**Usages**<br>B2.1 Maquettage & « prototypage rapide »<br>B2.2 Qualification d'une situation de pratique justifiant le besoin<br><br>**Marché**<br>B2.3 Quantification du besoin<br>B2.7 Identification de la proposition de valeur<br>B2.8 Analyse des attentes du marché<br><br>**Evaluation clinique**<br>B2.4 Identification du besoin médical<br>B2.5 Preuve de concept pré-clinique de principe d'action ou de mesure<br>B2.6 Identification des KOL	**Gestion de projet**<br>P2.1 Organisation du projet, planning et coopérations<br>P2.2 Première analyse du risque projet<br>P2.4 Identification des expertises requises (int, ext) et collège évaluateurs<br><br>**Réglementaire**<br>P2.5 Analyse des exigences générales en matière de sécurité et de performances<br>P2.6 Questionnement éthique du concept<br>P2.7 Conformité RGPD de la gestion de données du projet<br><br>**Financement**<br>P2.3 Préfiguration du business plan
**CML 3**<br>**Options, premières preuves de concepts, validation de briques**	**Développement technique**<br>T3.1 Simulation fonctionnelle dans le contexte<br>T3.2 Choix d'une solution technique et des modules logiciels<br>T3.4 Réalisation de maquettes pour chaque brique techno<br>T3.3 Levée des verrous technologiques<br>T3.6 Réalisation de POC en laboratoire (phantoms)<br><br>**Gestion des données**<br>T3.7 Structures de données logiciel<br><br>**Propriété intellectuelle**<br>T3.5 Brevets spécifiques	**Usages**<br>B3.3 Co-construction de scénarios d'usage adaptés<br>B3.2 Priorisation des tests d'usage<br>B3.1 Cas d'usages et analyse pluridisciplinaire des besoins<br>B3.8 Choix des caractéristiques prioritaires à intégrer dans les tests d'usage<br><br>**Marché**<br>B3.4 Positionnement produit et impact attendu<br><br>**Evaluation clinique**<br>B3.6 Stratégie clinique et premiers tests pré-cliniques<br>B2.9 Initiation du dossier d'évaluation clinique<br>B3.5 Confirmation du besoin médical	**Gestion de projet**<br>P3.3 Test beds (laboratoires d'essais)<br>P3.2 Mise à jour des éléments projet<br><br>**Réglementaire**<br>P3.1 Identification de la classe du DM et des contraintes réglementaires<br>P3.5 Première analyse du risque Produit<br><br>**Financement**<br>P3.4 Plan de financement du démonstrateur

FORUM LLSA

	Technologie	Besoin	Programmatique
**CML 4**<br>**Choix et définition du design, démonstrateur**	**Développement technique**<br>T4.1 Assemblage / intégration des briques technologiques pour la réalisation du démonstrateur matériel<br>T4.3 Démonstrateur logiciel assemblant tout le code et intégrant le protocole d'utilisation<br>T4.4 Prise en compte des risques techniques<br><br>**Gestion des données**<br>T4.2 Protection des données : confidentialité, cybersecurité<br><br>**Propriété intellectuelle**<br>T4.5 Vérification de liberté d'exploitation	**Usages**<br>B4.2 Vérification de l'usage médical avec les experts<br>B4.4 Caractérisation organisationnelle<br>B4.8 Evaluations UX/UI en laboratoire<br>B4.9 Evaluation formative d'utilisabilité en environnement simulé<br><br>**Marché**<br>B4.1 Priorités selon cible (marché) visée<br>B4.3 Chiffrage de l'impact attendu<br>B4.5 Fourniture des données de marché pour le business plan<br>B4.7 Validation du marché accessible selon les prérequis organisationnels<br><br>**Evaluation clinique**<br>B4.6 Essais cliniques préliminaires ou préclinique si réalisables<br>B4.10 Analyse des résultats des tests pré-cliniques<br>B4.11 Constitution du dossier d'évaluation clinique (suite)	**Gestion de projet**<br>P4.6 Identification des compétences complémentaires requises<br>P4.2 Définition du plan exploratoire d'investigation clinique et d'usages<br>P4.7 Actualisation : du risque projet, planning et budget en fonction du plan d'investigation clinique<br>P4.1 Premier cadrage du plan de développement produit<br><br>**Réglementaire**<br>P4.3 Analyse éthique du produit<br>P4.5 Analyse de risques Produit<br>P4.8 Initiation du dossier technique en vue du marquage CE<br><br>**Financement**<br>P4.4 Première formalisation du business plan
**CML 5**<br>**Développement de la solution stabilisée en vue de la validation clinique, pré-industrialisation**	**Développement technique**<br>T5.3 Fiabilisation et transfert technologique<br>T5.4 Conception (+gel), fabrication, pour prototype Alpha<br>T5.5 Développement du process de fabrication des proto.<br>T5.6 Gel des fonctionnalités et de l'IHM, fiabilisation de l'architecture d'intégration<br><br>**Gestion des données**<br>T5.1 Process de mise à disposition des data<br><br>**Propriété intellectuelle**<br>T5.2 Brevets d'amélioration / de fabrication / de process	**Usages**<br>B5.2 Définition des process et du schéma d'industrialisation des usages<br>B5.1 Vérification d'usage en environnement réaliste et contrôlé<br>B5.5 Partage et accompagnement avec les professionnels de la santé<br><br>**Marché**<br>B5.3 Market access strategy<br><br>**Evaluation clinique**<br>B5.4 Poursuite des essais précliniques et de leur analyse<br>B5.6 Constitution du dossier d'évaluation clinique (suite)<br>B5.7 Consultation précoce des autorités de santé concernant la démarche d'évaluation clinique	**Gestion de projet**<br>P5.7 Planification du dossier de marquage CE<br>P5.1 Partenariats de développement industriel<br>P5.5 Plan détaillé de développement de la solution<br>P5.2 Première analyse du risque process<br><br>**Réglementaire**<br>P5.4 Réalisation des essais réglementaires nécessaires à l'investigation clinique<br>P5.6 Activation du change control<br><br>**Financement**<br>P5.3 Recherche financement (SEED Capital)<br>P5.8 Préparation de la modélisation financière
**CML 6**<br>**Réalisation des essais cliniques en vue de l'obtention du marquage réglementaire**	**Développement technique**<br>T6.2 Analyse technologique du prototype Alpha pour amélioration des processus de production<br>T6.4 Production du lot de prototypes Beta<br>T6.5 Consolidation du dossier technique<br><br>**Gestion des données**<br>T6.1 Collecte, stockage, gestion et exploitation des données de l'étude clinique<br><br>**Propriété intellectuelle**<br>T6.3 Gestion de la PI relative aux données cliniques	**Usages**<br>B6.5 Evaluation sommative d'utilisabilité, d'acceptabilité (a priori), d'expérience utilisateur<br><br>**Marché**<br>B6.3 Caractérisation du dispositif sur la base d'enquêtes d'utilisation<br>B6.4 Définition du Minimum Viable Product<br>B6.7 Business plan implementation strategy<br><br>**Evaluation clinique**<br>B6.6 Rédaction du protocole clinique confirmatoire pour l'investigation clinique marquage CE<br>B6.1 Réalisation d'essais clinique(s) pivot(s), analyse des résultats et rédaction du rapport d'étude	**Gestion de projet**<br>P6.2 Mise à jour des éléments projet et risques<br><br>**Réglementaire**<br>P6.1 Cadre réglementaire lié à l'utilisation des données<br>P6.5 Consolidation du dossier technique (dépôt CPP-ANSM)<br>P6.6 Documentation utilisateur<br><br>**Financement**<br>P6.3 Implémentation de la modélisation financière<br>P6.4 Minimum viable Business Model(s)

FORUM LLSA

	Technologie	Besoin	Programmatique
**CML 7 Industrialisation, mise à l'échelle des moyens de production**	**Développement technique**<br>T7.1 Industrialisation<br>T7.2 Qualification chaîne de production et réalisation préséries<br>T7.3 Automatisation des tests de fonctions et reproductibilité logicielle<br><br>**Gestion des données**<br>T7.4 Définition des accès serveurs de données<br><br>**Propriété intellectuelle**	**Usages**<br>B7.2 Evaluations écologiques d'une présérie<br>B7.3 Analyse située de la perception du produit<br><br>**Marché**<br>B7.4 Eléments marketing (déploiement, export,..)<br>B7.5 Préparation de l'exportation<br><br>**Evaluation clinique**<br>B7.1 Poursuite des essais cliniques multi-centriques	**Gestion de projet**<br>P7.2 Révision des partenariats de développement industriel<br>P7.4 Identification des compétences marketing et commerciales<br><br>**Réglementaire**<br>P7.7 Essais réglementaires supplémentaires éventuels<br>P7.1 Dossier de marquage CE<br>P7.3 Preuve d'équivalence prototype par change control<br>P7.6 Vérification et planification des exigences de mise à jour du dossier technique<br><br>**Financement**<br>P7.5 Levée de fonds série A
**CML 8 Préparation de l'accès au marché**	**Développement technique**<br>T8.2 Ramp-up production<br>T8.3 Remontées et corrections de bugs logiciels et problèmes matériels<br><br>**Gestion des données**<br>T8.1 Mise en place des dispositifs de collecte de données<br>T8.4 Collecte et exploitation des données des études cliniques complémentaires<br><br>**Propriété intellectuelle**	**Usages**<br>B8.1 Tests d'usage en vie/environnement réel(le)<br>B8.5 Etude de l'acceptabilité sociale<br>B8.6 Etude des impacts organisationnels<br>B8.8 Etude complémentaire d'impacts organisationnels pour Article 51<br>B8.9 Etude complémentaire des impacts organisationnels<br><br>**Marché**<br>B8.2 Promotion, publicité, rencontre des promoteurs et KOL<br>B8.4 Mise en place de stratégies de communication à partir d'étude des usages<br>B8.10 Actualisation des hypothèses éco. avec les données de vie réelle<br>B8.11 Affinage des stratégies de go to market par typologie client<br><br>**Evaluation clinique**<br>B8.3 Réalisation et analyse des résultats des essais cliniques complémentaires, rapport d'étude<br>B8.7 Etudes cliniques complémentaires incluant études médico-économiques<br>B8.12 Rédaction du protocole clinique complémentaire	**Gestion de projet**<br>P8.1 Clôture projet<br><br>**Réglementaire**<br>P8.5 Dossier d'obtention du remboursement<br>P8.2 Prise en compte du cadre réglementaire pour la promotion de la solution<br>P8.6 Consolidation du dossier technique pour études complémentaires<br>P8.7 Cadre réglementaire lié à l'utilisation des données (études complémentaires)<br>P8.8 Dossier d'obtention du remboursement (études complémentaires)<br><br>**Financement**<br>P8.3 Levée de Fonds série A ou B<br>P8.4 Mise à jour des hypothèses économiques avec les données de vie réelle<br>P8.9 Levée de Fonds série A ou B (études complémentaires)
CML 9 Suivi post market, suivi en vie réelle du produit	**Développement technique**<br>T9.1 Production en série<br>T9.2 Gestion du cycle de vie du produit<br><br>**Gestion des données**<br>T9.3 Production de données de matério-épidémiologie<br>T9.5 Collecte et traitement des données en vie réelle<br><br>**Propriété intellectuelle**<br>T9.4 Veille concurrentielle et gestion des contrefaçons	**Usages**<br>B9.1 Poursuite des tests d'usage en vie réelle<br>B9.3 Suivi qualité PREM<br>B9.4 Analyse des modes d'appropriation par l'usager<br><br>**Marché**<br>B9.6 Commercialisation et vente du produit sur différents marchés<br><br>**Evaluation clinique**<br>B9.2 Suivi qualité PROM<br>B9.5 Etudes cliniques complémentaires (réévaluation prix, nouvelle indication, nouvelle revendication)	**Gestion de projet**<br>P9.2 Révision périodique des partenariats de développement industriel<br>P9.3 Préparation des prochaines générations<br><br>**Réglementaire**<br>P9.4 Matériovigilance et SCAC<br>P9.1 Renouvellement du marquage CE<br>P9.5 Mise à jour périodique du dossier technique<br><br>**Financement**
